# Interventions to improve uptake of urate-lowering therapy in patients with gout: a systematic review

**DOI:** 10.3399/bjgpopen20X101051

**Published:** 2020-07-08

**Authors:** Iqbal Gill, Nicola Dalbeth, Malakai 'Ofanoa, Felicity Goodyear-Smith

**Affiliations:** 1 Faculty of Medical & Health Science, University of Auckland, Auckland, New Zealand; 2 Bone and Joint Research Group, Faculty of Medical and Health Science, The University of Auckland, Auckland, New Zealand; 3 Pacific Health, The University of Auckland, Auckland, New Zealand; 4 Department of General Practice & Primary Health Care, The University of Auckland, Auckland, New Zealand

**Keywords:** gout, hyperuricemia, systematic review, primary health care, general practice

## Abstract

**Background:**

Gout treatment is suboptimal despite available therapy, with low levels of initiation and persistence of urate-lowering therapy (ULT) in many patients.

**Aim:**

To identify all interventions that have attempted to improve the uptake of ULT and analyse the clinical outcomes.

**Design & setting:**

A systematic review of international articles published in English.

**Method:**

A systematic search was conducted through MEDLINE, Embase, CINAHL Plus, and Scopus databases to identify all studies on relevant interventions for gout. Interventions were included if they aimed to address patient adherence with serum urate (SU) level as an outcome. This included patient education, practitioner monitoring, medication titration, SU monitoring, and ongoing patient engagement and follow-up. Follow-up studies to original interventions and those with only an abstract available were included.

**Results:**

Twenty articles met the inclusion criteria, describing outcomes of 18 interventions conducted in primary care settings: six nurse-led, five pharmacist-led, and seven multidisciplinary, multifaceted interventions. Improvement in SU levels was observed in all interventions. Nurse-led interventions were effective at empowering patients as they addressed illness perceptions and provided education, advice, and telephone follow-up. Pharmacist-led interventions primarily aimed to monitor patients, alter medication dosage, and provide automated telephone follow-up. Various multifaceted programmes involving a range of providers resulted in increased sustained use of urate-lowering medication.

**Conclusion:**

A nurse-led approach focusing on patient understanding about gout is the most effective in achieving improved patient adherence, and lowered SU levels among patients. An intervention should include patient education and follow-up components.

## How this fits in

Inadequate prevention of gout flares from the use of ULT is a global concern and leads to poor patient outcomes. This article systematically reviews interventions extending beyond the usual care provided by primary care clinics to improve ULT uptake. These often involve teamwork between nurses, pharmacists, and other providers assisting with the ongoing monitoring and medication adjustments. Nurse-led interventions appear to be the most effective. This is likely to be because these interventions involve the most patient engagement, which empowers patients to share decisions about their care and make the sustained behavioural changes required to take long-term medication.

## Introduction

Gout is a common form of arthritis caused by hyperuricaemia and subsequent deposit of monosodium urate (MSU) crystals in the joints. Gout flares are painful and cause disruption to employment and social activities. Gout also has other long-term sequelae, including formation of tophi and joint damage. Gout flares are typically treated with non-steroidal anti-inflammatory drugs (NSAIDS), colchicine, and/or corticosteroids. However, clinical guidelines recommend prevention of recurrent gout flares through treating the cause, with the use of medications such as allopurinol, febuxostat, and probenecid, which lower urate levels.^1,2^ Achieving and maintaining a target SU level of <6.0 mg/dl (0.36 mmol/l) eventually leads to dissolution of MSU crystals and prevention of gout flares.^3^

Inadequate treatment with ULT leading to poor patient outcomes is a global concern. A US study found that only 29% of patients with gout were on ULT, and only half of these had achieved the target SU.^4^ Studies in the UK show that only 34% to 38% of patients with gout get initiated on ULT, and only 39% of these persist with therapy after 1 year.^5,6^Another UK study found only 32% of patients with gout were prescribed ULT.^7^ In Aotearoa/New Zealand (A/NZ), Māori and Pacific peoples have a higher prevalence of gout (for example, 22% of Pacific men aged >20 years) than other ethnic groups, but only 35% of Pacific people and 40% of Māori people with gout receive continuous ULT, compared with 44% of non-Māori and non-Pacific peoples.^8^

Reasons for low ULT initiation and persistence rates include physicians not offering the treatment, and patients choosing not to take it, as this therapy requires clinician and patient persistence, whereas treating acute symptoms gives rapid results for patients. Patients need to have repeated measures of their urate levels in order for their allopurinol dose to be titrated. Deficiencies in ULT management include lack of adequate monitoring, failure to treat to the SU target, and hesitancy to increase ULT in patients with concurrent conditions such as chronic kidney disease.^9^ Patient understanding about the rationale and the need for long-term medication use is important to support adherence to a long-term medication.^10,11^

The aim of this review was to identify interventions implemented to address low ULT uptake internationally, both those that proved to be effective and those that were not effective. The objective was to analyse the characteristics of these interventions to inform potential future gout management programmes.

## Method

### PICOS process

The ‘PICOS’ process was used to develop the plan for the literature search. The Population in question was patients with gout; the Intervention was any management strategy designed to increase ULT uptake; Comparison with control, other interventions, or before–after studies were included; Outcomes are discussed under variables sought; and Study designs included randomised controlled trials, quality improvement projects, observational studies, and qualitative research.

### Search strategies

The reporting of this study follows the Preferred Reporting Items for Systematic Reviews and Meta-Analyses (PRISMA) guidelines (see Appendix 1). The search terms used were ‘gout’, ‘urate’ or ‘uric acid’ or ‘allopurinol’ and ‘intervention’ or ‘management’ or ‘self-management’. Databases searched were MEDLINE (Ovid), Embase (Ovid), CINAHL Plus, and Scopus, augmented by grey literature and hand searches. The initial search and screening was conducted by IG and subsequently checked by a senior researcher, FG. See Appendix 2 for the electronic search strategies.

### Inclusion criteria

Non-English articles were excluded but there was no limit on year of publication. All studies until September 2019 were included. All research aimed at increasing ULT uptake were included, including pilot, full trial, and follow-up studies. All types of intervention were included: educational, nurse-led, pharmacist-led, and technology-based applications.

**Figure 1.**
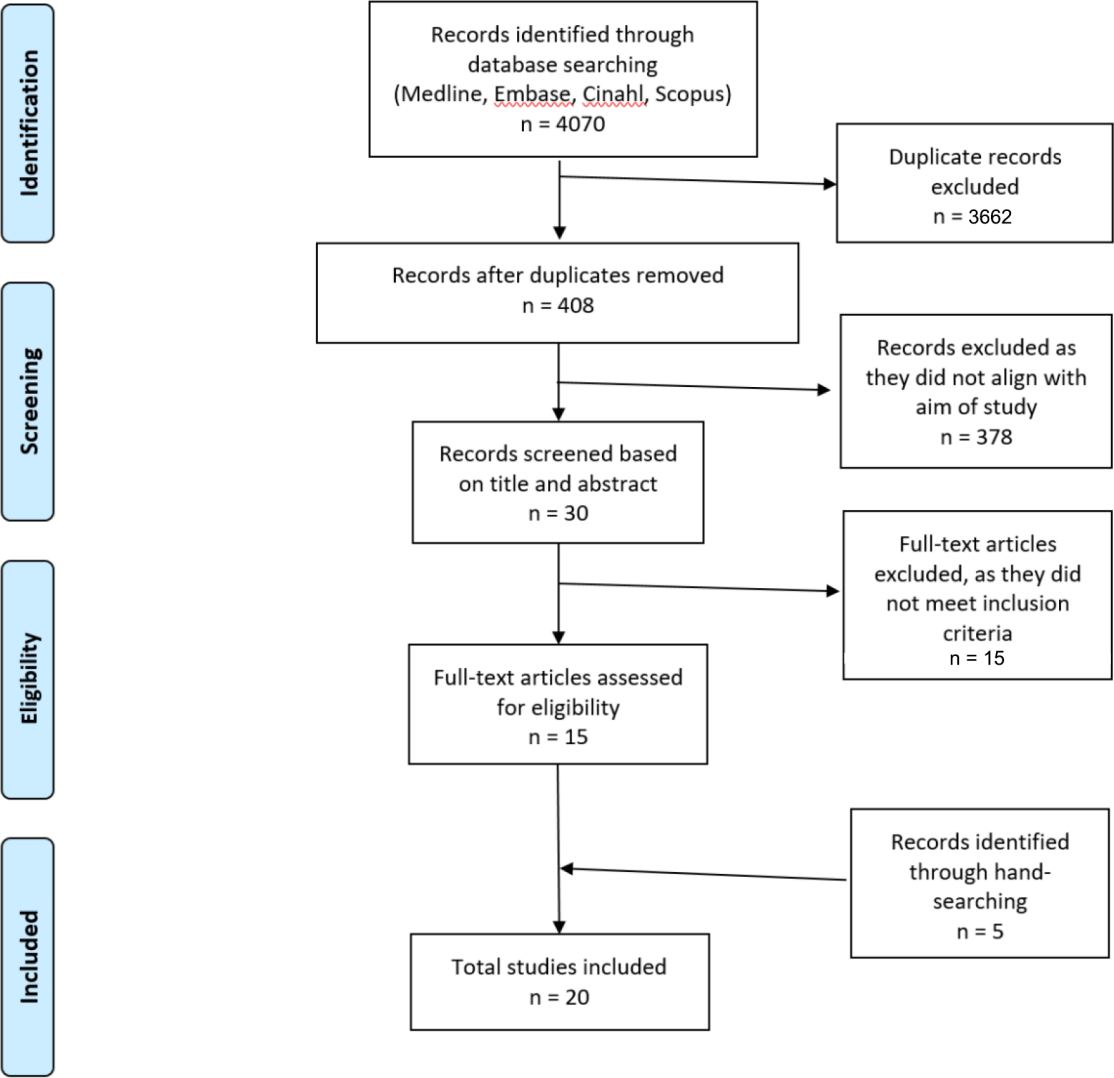
PRISMA flowchart of the selection process From, Moher *et al*.^44^

The PRISMA strategy was used to identify the studies, remove duplicates, screen on title, determine eligibility from the abstract, and then to review the full-text article to decide whether the study was to be included. A PRISMA flowchart was produced (Figure 1).

### Variables sought

The data extracted from the research included the study design (for example, randomised controlled trial, observational study, evaluation study, quality improvement project, qualitative research), the nature of the intervention (for example, nurse-led, pharmacy-led, patient education, telemedicine), its duration, and number of participants. Outcome measures were as follows: changes in ULT use or adherence; changes in SU or time to reach target level; knowledge measures; rate of gout flares; presence of tophi; hospitalisations owing to gout; quality of life; and perceived patient usefulness of intervention.

### Analysis

Data from the selected studies were extracted, tabulated, and synthesised narratively. The heterogeneity of the studies precluded meta-analysis and assessment of study quality. The usual data measure was significant difference (*P* value). Given this was a systematic review of previously published studies, this research was exempt from international review board approval.

## Results

A total of 4070 (678 MEDLINE, 1685 Embase, 345 CINAHL Plus, 1362 Scopus) articles were retrieved, augmented by hand-searching. After assessing for eligibility, 20 articles met the inclusion criteria, describing outcomes of 18 interventions; six nurse-led, five pharmacist-led, and seven multidisciplinary, multifaceted interventions (Figure 1).^12–31^ The characteristics of the nurse-led, pharmacist-led, and other intervention studies are recorded in Tables 1–3 respectively.

**Table 1.** Characteristics of included nurse-led studies

**Author,** **year, country**	**Design**	***N***	**Duration, months**	**Details of intervention**	**Outcomes**
Murphy and Schumacher, 1984US^^24^^	RCT	63	24	Randomly assigned to four educational groups:A: education by rheumatology fellowB: pamphlet on goutC: intensive education by nurseD: monthly telephone call from nurse	Mean SU levels:No change A & B (initial: 8.3 mg/dl to final: 8.2 mg/dl). Improved C & D (initial: 8.8 mg/dl to final: 6.4 mg/dl)
Lim *et al*, 2012Singapore^^21^^	QIP	126	12	Comprehensive patient education, enhanced telephone access for nurses to call patients, reappointments and refills, increased frequency of visits until target SU concentration achieved	Median time to achieve target SU (<6 mg/dl) was 36.9 weeks with 72% of patients achieving that by 12 months
Rees *et al*, 2013UK^^25^^	Observ	106	12	Education, individualised lifestyle advice, appropriate ULT	92% achieved therapeutic target (<6 mg/dl after 1 year)
Abhishek *et al*, 2017UK^^12^^	Observ	75	60	Questionnaire sent to 100 patients with gout from previous study^25^ to examine adherence of ULT by nurse-led care	5-year persistence on ULT was 91%. Of these, 85% had SU level <5 mg/dl after 5 years
Yoo *et al*, 2017South Korea^^28^^	RCT[Abstract]	100	3	Patient education randomly assigned to education and non-education groups. Intervention group received nurse-led face-to-face education. Both groups: information leaflet, and ULT	Increased knowledge, improved drug compliance, target SU decreased (5.73 cf 6.08 mg/dl) in education versus non-education group
Tay *et al*, 2017Singapore^^27^^	Observ [Abstract]	100	9	Telemedicine bundle: nurse education, six 2-weekly phone calls, hotline; 6-weekly lab tests and couriered ULT	Median time to achieve target SU levels 19 weeks, no hospitalisation gout flares
Doherty *et al*, 2018UK^^14^^	RCT	517	24	Randomly assigned to nurse-led care educating patients and involved in management decisions or usual care by GPs	Nurse versus GP groups at 2 years:SU levels <6 mg/dl 95% cf 29%. 88% cf 16% SU <5 mg/dl.Presence of tophi r3% cf 11% (*P*<0.0001)Risk of flares 8% cf 25% (*P*<0.0001)Quality of life SF-36 physical component 41 cf 37 (*P*<0.0001)
Latif *et al*, 2019UK^^19^^	Qual	30	4	Participants involved in observational study^25^ interviewed to explore patient perception of nurse-led care	Nurse-led cf to GP care reported improved knowledge and understanding of ULT, leading to long-term adherence to ULT by patients

Cf = Compare. Observ = observational. Qual = qualitative. QIP = quality improvement project. RCT = randomised controlled trial. SU = serum urate. ULT = urate lowering therapy. Vs = versus. Wk = week.

**Table 2.** Characteristics of included pharmacist-led studies

**Author,** **year, country**	**Design**	***N***	**Duration, months**	**Details of intervention**	**Outcomes**
Goldfien *et al*, 2014US^^17^^	Observ	100	11	Pharmacist+ rheumatologist provided education by phone; employed standard gout medication to lower SU levels	SU levels ≤6 mg/dl achieved and maintained at least 3 months for 78/95 patients
Goldfien *et al*, 2016US^^16^^	RCT	77	6	Telephone-based programme to manage SU. Implementation of protocol, adjusting standard gout management; patient adherence monitored	SU levels at ≤6.0 mg/dl at 26 weeks in 35% of intervention cf 13% control group
Whiteman *et al,* 2018UK^^31^^	Observ [Abstract]	52	23	Education about gout and its treatment. Offered ongoing monitoring until SU at target	38/52 patients discharged from service. Average SU level decreased from 7.7 mg/dl to 4.9 mg/dl
Mikuls *et al*, 2019US^^32^^	RCT clustered	1,412	24	Randomised by site. Intervention telephone interactive voice recognition system to assess adherence, encourage SU and other lab monitoring, provide patient-focused gout education, and adjust allopurinol dosing versus usual care	Better adherence to ULT 50% cf 37% usual care. SU <6 mg/dl intervention 30% cf 15% usual care
Huang *et al*, 2019US^^18^^	QIP	36	12	19 referred patients received pharmacist education and ULT titration programme cf with 28 non-referred	SU improved intervention (8.8–6.1 mg/dl for intervention cf 7.6 to 6.8 mg/dl for usual care. 32% intervention and 25% usual care achieved target

Cf = Compare. Observ = observational. Qual = qualitative. QIP = quality improvement project. RCT = randomised controlled trial. SU = serum urate. ULT = urate lowering therapy. Vs = versus. Wk = week.

**Table 3.** Characteristics of included studies on other interventions

**Author,** **year, country**	**Design**	***N***	**Duration, months**	**Practitioners involved**	**Details of intervention**	**Outcomes**
Leyva *et al*, 2013UK^^20^^	QIP[Abstract]	13	3	Physician, rheumatology clinic	Patient goalsetting; continuous reinforcement via weekly phone calls	10/13 reduced SUA, 80% reached SU goal ≤6 mg/dl
Moffat and McNab, 2015US^^23^^	QIP	126	12	GPs and admin staff	Practice-based audit of SU levels, ULT titration, lifestyle advice last 12 months. Target SU <6 mg/dl	112 (84%) SU level checked; 79 (51%) reached SU <6 mg/dl in 6 months
Fields, *et al* 2017US^^15^^	Observ	40	12	Nurse and pharmacist	Gout patient self-management Knowledge exam Nurse-taught curriculum Monthly phone calls from pharmacists	84.6% reported increased knowledge; 81% found nurse education useful, 50% found pharmacist phone calls useful
Callear *et al*, 2017UK^^30^^	QIP	115	12	Primary care team	Improvement cycles with patient education and increased SU monitoring	Reduced SU (0.37 to 0.3 mg/dl); 20% reduction in gout flares; improved compliance 63% to 91%
Bulbin *et al*, 2018US^^13^^	QIP	819	6	Primary care providers and rheumatology staff	Two practices: one intervention, one usual care. Electronic tool to identify undertreated patients, assist telephone encounters, reminders, request tests, provide advice	Intervention improved 54% to 61%; patients monitored improved from intervention patients treated 56% to 79%; reached SU target ≤6.0 mg/dl 27% to 43%. Control no change
Lawrence *et a* *l*, 2019A/NZ^^29^^	Open evaluation	887	3	Gout educators and pharmacist monitoring	Free blister-pack medication, recall for SUA, result communicated to prescribers	44% completed programme and reached SU target ≤0.36 mmol/l
Stamp *et al*, 2019A/NZ^^33^^	Open evaluation	171	Pre-interven audit 24; post interven 12	Rheumatologist, nurse specialists, practice nurses	Multidisciplinary education; structured approach to treating gout flares and ULT; screening; telephone consultations available; electronic prompts; recall system; gout treatment template	Reduction in not reaching target SU 52/133 (39%) cf 43/67 (64%) in 2012 *P* *<* *0.001*. Increase in average SU test 2 (0–10) cf 1 (0–3) in 2012 *P* *<* *0.001*.

Admin = administrative staff. A/NZ = Aotearoa/New Zealand. Cf = Compare. Interven = intervention. Observ = observational. QIP = quality improvement project. RCT = randomised controlled trial. SU = serum urate. ULT = urate lowering therapy.

The studies were conducted in the UK, US, A/NZ, South Korea, and Singapore. There are five randomised controlled trials (RCT),^14,16,24,28,32^ six observational studies,^12,15,17,25,27,31^ six quality improvement projects or pragmatic intervention studies,^13,18,20,21,23,30^ two open evaluation studies,^29,33^ and a qualitative study.^19^ The latter was a subsequent investigation of an observational study^25^ that also had a 5-year cross-sectional follow-up.^12^ For three studies, only a published conference abstract was available.^27,28,31^ All study participants were patients with gout in primary care or rheumatology outpatient settings, and the duration of follow-up ranged from 3 months to 5 years, with 11 for at least 1 year.

### Nurse-led interventions

Eight articles involved six nurse-led interventions.^12,14,19,21,24,25,27,28^ One of the observational studies^25^ had an additional article exploring perceptions of participants,^19^ and a subsequent 5-year follow-up study^12^ (Table 1). Interventions were multifaceted, including assessing the patient’s beliefs and perceptions about the illness and its management, patient education, telephone reminders for assessing SU levels and prescription refills and monitoring until the target SU was reached. Patient education covered a number of components such as information on the nature, causes, consequences, and treatment options of gout, and patient perceptions of illness, aimed at encouraging shared decision-making.^14^ Outcome measures included patient knowledge, change in SU levels, duration to reach target level, prescription rates of ULT, and hospitalisation rates for gout. All studies reported success such as improved achievement of SU targets in the intervention group compared with the usual care controls. Nurse-led care also resulted in improvements in gout flares, presence of tophi, and quality of life.^14^

### Pharmacist-led interventions

There were five studies where pharmacists led the interventions, which were generally providing patients with information plus monitoring of SU levels and ULT titration (Table 2).^16–18,31,32^ Pharmacist interventions were generally protocol-driven, rather than providing individual discussion and education with patients about their options.^16–18,32^ The largest trials use automated telephone systems rather than personal communications. Again, those receiving the interventions generally had improved SU levels and better adherence to taking ULT than those getting usual care.

### Multifaceted or multipractitioner interventions

A further seven had a multidisciplinary approach, with a variety of primary care staff — including physicians, nurses, pharmacist, administrative staff, and community educators — working together to provide different elements of the intervention.^13,15,20,23,29,30,33^ These involved different combinations of patient engagement (goal-setting, education), and systematic approaches to reminders, recalls, monitoring, and repeat prescribing. Processes were assisted by electronic tools and prompts in some instances.^13,33^ In one study involving both nurse education and pharmacist phone-call monitoring, participants rated the input from the nurse as more useful than that from the pharmacist.^15^ None of these studies were RCTs, but in all cases the intervention led to improved measures from either baseline or a usual care control group.

### Patient perspective

A major component in ULT uptake is patients understanding the value to them of this approach and why blood tests are needed to determine the medication dose they take, as well as making it easier for them to comply with requirements. Very few studies examined the patient perspective, assessing which components of interventions were successful. A study that involved both nurses and pharmacists assessed the usefulness of the programme in understanding and managing their gout from patients’ points of view.^15^ Eighty-one per cent rated as helpful the education provided by the nurses, 55% the monthly phone calls from pharmacists, and 85% a combination of these interventions given together. The authors noted that developing an action plan, ongoing education, and monitoring were all important components, but where a multidisciplinary team is not available, these roles might be played by a single health provider.

The qualitative study^19^ conducted 18–26 months after a nurse-led intervention^25^ found that nurse education increased patient confidence in consistently taking maximised ULT compared with the fixed, low-dose ULT they had been receiving from their primary care practitioners, after they realised that the dosage needed to be optimised. Furthermore, the availability of information regarding their SU levels provided psychological motivation to continuously receive ULT, increasing persistence. Nurse-led interventions were more patient-centred; hence, they enabled patients to have greater involvement in decision-making, as they were able to communicate their concerns, and weigh the benefits and risks of treatment options with the nurse.

## Discussion

### Summary

All studies reported successful increases in the uptake of ULT, although effect size was variable. The nurse interventions, which involved patient-centred approaches, had a much larger effect on medication persistence, SU target, and important clinical outcomes such as flare rate, than the pharmacy-led ones. Only five were RCTs, and many had small sample sizes and short durations of evaluation that limit generalisability. Generally the studies fail to provide insight into the long-term effectiveness of these programmes, with the exception of a follow-up study that showed 85% had sustained low SU levels 5 years after a nurse-led intervention.^12^

Some of the improvements noted may be due to the Hawthorne effect, whereby change in patient behaviour is influenced by their awareness of being observed.^34^ Participants are more likely to respond to treatments and increase ULT adherence when they know they are in a study, and they may have already been motivated to improve their SU levels prior to the intervention and hence agreed to participate.^13^

### Strengths and limitations

This work systematically searched multiple databases, including grey literature, unrestricted by date or study type. The search and selection strategies were checked by a senior researcher. However, studies included a range of methodologies, including quality improvement projects, which limit generalisability, and quality scores were not assigned to each study. Moreover, three research abstracts without full-text articles were included. The search was limited to English, which might have omitted eligible articles published in other languages.

### Comparison with existing literature

This is the first systematic review on the effectiveness of interventions to improve the uptake of urate-lowing medications in primary care settings.

There has been a strong move towards patients’ active involvement in managing their own health. Self-management programmes need to address medical, behavioural, and emotional management.^35^ Drawing from Social Learning Theory,^36^ there has been a progression of models looking at behavioural change since the 1980s. The Health Belief Model^37^ looks at factors leading to 'readiness to act', and led on to Prochaska’s Spiral Model of Stages of Change,^38^ which describes the dynamics of behaviour change from pre-contemplation through contemplation to preparation to action and maintenance.

Other models look at how clinicians can assist patients to change. The Five A’s Behaviour Change Model^39^ involves: *assessing* patients’ level of behaviour, beliefs and motivation; *advising* them based on their personal health risks; *agree*ing with them on a realistic set of goals; *assisting* to anticipate barriers; developing a specific *action* plan; and *arranging* follow-up support. This can be combined with the ‘5Rs’ model applied to motivate patients: asking why making the behaviour change is personally *relevant*; identifying the *risks* of continuing with the unhealthy behaviour; looking at the *rewards* for making changes; identifying the barriers or *roadblocks* that might impede making the change; and *repeating* this motivational interview every time the patient consults.^40^

Interventions to increase ULT use require patients to understand the importance of taking action, accessible means to monitor their SU levels, and ongoing input and support from their health provider so that behaviour changes are sustained.

### Implications for research and practice

Collectively, this work provides support for interventions that focus on patient understanding and adherence to ULT. The largest effects have been reported with intensive nurse-led gout management, delivered by research nurses in a primary care setting. While this intervention has been shown to be effective in a clinical trial setting in the UK, it is unclear how such an approach should be implemented more broadly within primary care. In particular, standardisation of the intervention, workforce training, and cost to patients in other healthcare systems requires careful consideration. Many of the interventions delivered to date are multifaceted, and it is uncertain at present which aspects contribute most to their success.

In A/NZ, rates of gout are high in Pacific and Māori populations, particularly in men.^8^ Forty-seven per cent of Pacific men aged >65 years have gout, compared with 17% of non-Pacific and non-Māori men. Pacific people have earlier onset, higher flare frequency, more joint inflammation, greater hospitalisation rates, and lower health-related quality of life than non-Pacific people.^41^ Māori rates fall between Pacific and non-Pacific non-Māori. Despite recognition of this undertreatment,^41^ use of ULT to prevent gout flares remains low and static.^42,43^

Research priorities are to design and implement effective interventions to increase ULT, which are tailored to Pacific and Māori populations to reduce the primary care workload relating to managing untreated gout, and result in considerable health gains for these populations. The authors are planning a co-design approach, combining knowledge from this review with brainstorming ideas from a variety of relevant stakeholders as to the contexts in which interventions should be delivered and the strategies that should be used to engage the local communities.

In summary, the interventions included a number of different activities, extending beyond the usual care provided by primary care clinics to improve the uptake of ULT. Teamwork between nurses and pharmacists, and other providers assisted with the ongoing monitoring and medication adjustments. However, nurse-led interventions appear to be the most effective. This may be because they involve the most patient engagement, empowering patients to share decisions about their care, which increases the likelihood that they will make the sustained behavioural changes required to take long-term medication.

## Notes

Dr Dalbeth reports grants and personal fees from AstraZeneca, grants from Amgen, personal fees from Dyve, personal fees from Hengrui, personal fees from Horizon, personal fees from Kowa, personal fees from Abbvie, personal fees from Pfizer, personal fees from Janssen, outside the submitted work. No other author has any conflict of interest to declare.
